# Morphological features of the chiasma tendinum and its relation with surface landmarks and pulleys: a cadaveric study

**DOI:** 10.1007/s00276-021-02783-w

**Published:** 2021-07-01

**Authors:** Uğur Dinç, Ecem Şengezer, Orhan Beger, Merve Şehide Yılmaz, Zeliha Kurtoğlu Olgunus

**Affiliations:** 1 Faculty of Medicine, Justus Liebig University , Gießen, Germany; 2 Department of Anatomy, Faculty of Medicine, Mersin University, Mersin, Turkey; 3 Faculty of Medicine, Mersin University, Mersin, Turkey; 4 Department of Family Medicine, Sisli Etfal Training and Research Hospital, Istanbul, Turkey; 5 Department of Anatomy, Faculty of Medicine, Gaziantep University, Gaziantep, Turkey

**Keywords:** Camper chiasm, Flexor tendons, Tendon sheath, Tendinous chiasm, Hand

## Abstract

**Aim:**

Chiasma tendinum (Camper’s chiasm) is of great importance in the delicate movements and stability of the fingers and takes place poorly in the literature. This study aims to reveal the morphometric details of the chiasma tendinum in relation with pulleys and other relevant structures.

**Materials and methods:**

Palm and 2nd to 5th fingers of 10 (6 male, 4 female) formalin fixed cadavers were used bilaterally. After determining the superficial reference points on the fingers, the skin and the tendon sheath were incised, and then measurements of chiasma tendinum and related tendons were performed. The measurements were analyzed with respect to fingers, genders, and sides. Finally, the types of chiasma tendinum were identified and then grouped as symmetrical, asymmetrical, and pseudo chiasm.

**Results:**

Pulley and chiasma tendinum positions were correlated with finger length (*p* < 0.01). Pulley lengths were significantly less in females. Asymmetrical chiasma tendinum types were found in 45% of the fingers. In most comparisons, values for fifth finger were significantly different than that of other fingers and chiasma tendinum types differed according to fingers and gender. The case of no fiber exchange was observed only in the 5th finger in 15%.

**Conclusion:**

Findings related to the prediction of location of the pulleys and chiasma tendinum according to the superficial signs, awareness of cases where one of the two arms of the flexor digitorum superficialis is extremely thin and no fiber exchanges that may be risk factors for spontaneous tendon rupture may help provide more accurate approaches in relevant clinical applications.

## Introduction

Tendons of the flexor digitorum superficialis (FDST) continue inside the tendon sheaths of 2nd–5th finger and before attachment, each tendon of each finger splits into two branches to form a chiasm, finally each arm inserts to the anterior aspect of medial phalanx. Four tendons of the flexor digitorum profundus (FDPT) insert to distal phalanx without further division and by passing between the two arms of FDST [16, 19, 21]. In each finger, two arms of the FDST exchange fibers before attachment, which is called chiasma tendinum (CT), tendinous chiasm, or Camper’s chiasm [16, 19]. Variations are common, and were studied by Schmidt et al. and Gonzalez et al. [7, 18].

Certain thickened parts of the tendon sheath surrounding the FDST and FDPT are known as pulleys. The fact that the fingers are highly mobile requires the support and flexibility functions provided by the 5 annular pulleys (named as A1, A2, A3, A4, and A5) and 3 cruciform pulleys (named as C1, C2, and C3). Annular pulleys are stronger and thicker (support function), whereas cruciform pulleys are more flexible (elasticity function) and situated at the flexion points [16, 19, 21]. Although there are numerous studies on pulleys in the literature, the data analyzing the relationship of pulleys with both superficial landmarks and CT are scant.

Surgical importance of the pulleys and CT are due to their locations in the tender zone 2. Zone 2, known for its inadequate arterial supply, was called “no man’s land” until modern techniques were developed and surgeries in the area were avoided [1, 4, 15, 21] The details of CT are of surgical importance, as some surgical procedures involve direct manipulation of the flexor tendons, such as reconstructing the A2 pulley using an arm of the CT [21]. It is known that A2 and A4 pulleys should be protected to avoid “bowstring” in hand surgeries [10, 21]. The relative and absolute positions of the pulleys and other related structures can contribute to the effectiveness of such surgeries and guide surgery planing. Inconsistencies in the limited number of studies in the literature on CT morphometry and distribution of its types [7, 18] point to the need for further studies.

The aim of this study is to provide data on the morphometry of CT and pulleys, and distribution of CT types in each finger to understand the structural background of clinical and functional variations in individuals. In addition, parameters describing the position of the CT and pulleys according to the superficial landmarks of the hand will help to estimate the morphometrical features of the structures in deep layers in a narrow surgical area such as finger.

## Materials and methods

The study was approved by the Board of Ethics of Mersin University (approval number is 2018/337). We dissected the upper extremities of 10 formalin fixed cadavers (6 male, 4 female; aged between 45 to 86 years) in the inventory of Mersin University, Faculty of Medicine, Anatomy Department Laboratory. We used dissection microscope.

(Carl Zeiss, Carl Zeiss Meditec AG, Germany) digital camera (Nikon D5200 18–105 mm) and digital caliper with 0.01 mm sensitivity (MARCAL 16 ER, Mahr, Gottingen, Germany).

The reference points for measurements were set out in three layers. Before dissections, measurements for superficial landmarks (palmar creases) were completed. Secondly, after longitudinal incision of the skin, the pulleys were exposed and their proximal and distal borders were marked with colored pins and the placement and width measurements of pulleys were completed. For the third layer of dissection, the tendon sheath was cut longitudinally. According to the classification modified mainly from Schmidt et al. [18], the CT type was determined, and then the reference points for CT measurements were carried out.

The list of parameters with their abbreviations are as follows:

### **A. Relation of superficial landmarks (creases) with finger tip**

FT-DPC: fingertip–distal interphalangeal crease distance;

FT-PPC: fingertip–proximal interphalangeal crease (in cases with one wide or double crease, the middle point was used);

FT-PDC: fingertip–palmar digital crease distance (in cases with one wide or double palmar digital creases, only proximal border was used);

FT-IPC: fingertip–interpalmar line distance (interpalmar line was defined as the line joining the ulnar origin of the distal palmar crease and the radial origin of the proximal palmar crease. [22]).

FT-WC: fingertip–wrist crease distance.

### **B. Placement of pulleys according to finger tips**

FT-A5D: fingertip–distal border of the A5 pulley (Since A5 pulley is quite short, no measurement was made from the proximal border);

FT-A4D: fingertip–distal border of the A4 pulley;

FT-A4P: fingertip–proximal border of the A4 pulley;

FT-A3D: fingertip–distal border of the A3 pulley (since A3 pulley is quite short, no measurement was made from the proximal border. This point was fixed with a pin before the tendon sheath was dissected.);

FT-A2D: fingertip–distal border of the A3 pulley (this point was fixed with a pin before the tendon sheath was dissected.);

FT-A2P: Fingertip–proximal border of the A2 pulley;

FT-A1P: Fingertip–proximal border of the A1 pulley;

FT-A1D: Fingertip–distal border of the A1 pulley.

### **C. Morphometry of pulleys**

A4L: A4 pulley length (distance between the distal and proximal borders of the A4 pulley);

A4T: A4 pulley thickness;

A3L: A3 pulley length (distance between the distal and proximal borders of the A3 pulley);

A2L: A2 pulley length (distance between the distal and proximal borders of the A2 pulley);

A2T: A2 pulley thickness;

A1L: A1 pulley length (distance between the distal and proximal borders of the A1 pulley);

C1L: C1 pulley length (distance between the distal and proximal borders of the C1 pulley);

C2L: C2 pulley length (distance between the distal and proximal borders of the C2 pulley);

C3L: C3 pulley length (distance between the distal and proximal borders of the C3 pulley).

### **D. Morphometry of radial and ulnar arm of FDST and FDPT**

FDST-RW: width of radial side of the two terminal tendons of FDST after CT.

FDST-RT: thickness of radial side of the two terminal tendons of FDST after CT.

FDST-UW: width of ulnar side of the two terminal tendons of FDST after CT.

FDST-UT: thickness of ulnar side of the two terminal tendons of FDST after CT.

FDPT-T: FDPT thickness was measured at the level of the proximal border of the A1 pulley.

FDPT-W: FDPT width was measured at the level of the proximal border of the A1 pulley.

### **E. Location of CT, radial, and ulnar insertions point of the FDST**

D1: distance from the proximal point of the CT to the proximal point of the ulnar insertion of FDST;

D2: distance from the proximal point of the CT to the proximal point of the radial insertion of FDST;

D3: distance from the proximal point of the CT to the distal point of the ulnar insertion of FDST;

D4: distance from the proximal point of the CT to the distal point of the radial insertion of FDST;

D5: distance between the proximal and distal points of the ulnar insertion of FDST;

D6: distance of the proximal and distal point of the radial insertion of FDST;

D7: closest distance between the radial and ulnar insertion points of the FDST;

D8: distance between the proximal and distal borders of the CT;

D9: width of the CT (measured as the transverse distance where fiber exchanges crossed each other; It was not measured for CT type 9G);

H-FDST R: proximal border of tendinous hiatus–radial insertion point of FDST (ulnar insertion was mostly parallel to radial insertion, thus only one (radial) insertion was chosen as distal end);

H-C: upper border of the tendinous hiatus–proximal point of the CT.

### **F. Relation of CT with A2 and A4 pulleys**

A2D-H: distal border of the A2 pulley–upper point of the tendinous hiatus;

A2D-C: distal border of the A2 pulley–proximal point of the CT;

A4P-H: proximal border of the A4 pulley–upper point of the tendinous hiatus;

A4P-C: proximal border of the A4 pulley–proximal point of the CT distance.

### **G. Classification of CT**

**Fig. 1:**
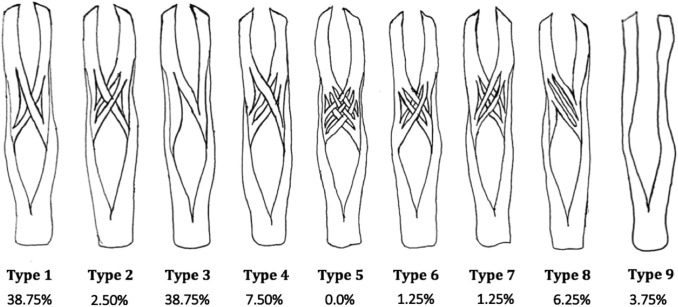
Distribution of CT types (a newly drawn combined and modified version of Schmidt et al. and Gonzalez et al.’s works)

Gonzalez et al. and Schmidt et al. defined chiasm classifications [7, 18]. In this study, mainly the classification of Schmidt et al. [18] was adopted, but only ‘type 9’ from the classification of Gonzales et al. was added [7]. The types in the classification are shown in Fig. 1.

### Statistical analysis

The parameters were compared between fingers (fingers 2,3,4, and 5), sexes (female and male) and sides (left-hand side and right-hand side). Each group (e.g., for comparisons between fingers, each of the four groups) was tested for normality with Shapiro–Wilk test or Kolmogorov–Smirnov test, depending on the dataset size before comparison. Differences among the fingers were evaluated by ANOVA, and then Bonferroni test was used for post hoc analyses. The difference between the genders was evaluated using Student’s *t* test for normally distributed groups and Mann Whitney *U* for non-normally distributed groups, while the difference between the sides was evaluated using the Paired *t* test and Wilcoxon test, respectively. Statistical significance threshold for comparison was taken as *p* < 0.05. Correlations among the parameters were analyzed by Pearson’s correlation test. Statistical significance threshold for correlation was taken as *p* < 0.01.

## Results

Descriptive statistics of parameters for each finger and corresponding ANOVA analysis results are shown in Table 1. Descriptive statistics and comparison for genders are shown in Table 2. The main findings are summarized below.

**Table 1:** Descriptive statistics of the measurements [mean ± SD or median (25th–75th percentiles)] and the results of ANOVA

Main topic	Parameters	Finger 2 (mm)	Finger 3 (mm)	Finger 4 (mm)	Finger 5 (mm)	*p*
A. Relation of superficial landmarks (creases) with finger tip	FT-DPC	24.03 ± 2.44	25.10 ± 2.22	24.83 ± 2.17	22.76 ± 2.04 ^bc^	< 0.01
	FT-PPC	40.39 ± 3.74	43.71 ± 4.69	43.40 (34.39–72.79)	35.78 ± 3.82 ^abc^	< 0.01
	FT-PDC	59.66 ± 5.88	64.59 ± 5.87	60.80 ± 4.92	50.72 ± 6.87 ^abc^	< 0.01
	FT-IPC	82.51 ± 8.12^b^	92.71 ± 9.45	87.98 ± 7.45	70.51 (57.33–102.75) ^abc^	< 0.01
	FT-WC	151.03 ± 14.60	159.73 ± 14.14	148.51 ± 13.16	127.04 (57.18–166.56) ^abc^	< 0.01
B. Placement of pulleys according to fingertips	FT-A5D	17.32 ± 3.38	18.26 ± 2.93	18.80 ± 3.70	15.29 ± 2.89 ^bc^	< 0.01
	FT-A4D	26.00 ± 3.40^bc^	29.21 ± 2.80	29.35 ± 2.96	23.20 ± 3.45 ^abc^	< 0.01
	FT-A4P	34.54 ± 2.94	36.93 ± 3.12	36.93 ± 3.16	30.32 ± 4.12 ^abc^	< 0.01
	FT-A3D	41.59 ± 4.11^bc^	45.87 ± 4.49	45.14 ± 3.75	35.43 ± 3.53 ^abc^	< 0.01
	FT-A2D	53.05 ± 5.84	57.66 ± 6.85	57.11 ± 4.87	46.55 (30.82–70.18) ^abc^	< 0.01
	FT-A2P	71.57 ± 8.88^b^	79.55 ± 8.35	75.91 ± 6.98	60.30 (26.60–73.82) ^abc^	< 0.01
	FT-A1P	77.52 ± 10.64^b^	88.92 ± 9.41	83.78 ± 10.66	66.90 ± 7.75 ^abc^	< 0.01
	FT-A1D	74.20 ± 9.06^b^	81.85 ± 8.40	76.16 (69.85–99.87)	64.60 ± 6.97 ^abc^	< 0.01
C. Morphometry of pulleys	A4L	8.36 (4.73–16.49)	8.80 ± 2.31	7.96 ± 1.41	6.75 ± 1.49 ^ab^	< 0.01
	A4T	0.40 ± 0.16	0.46 ± 0.14	0.45 ± 0.16	0.36 (0.12–0.87)	0.66
	A3L	2.67 ± 0.62	3.01 (2.57–5.45)	3.18 ± 0.97	2.87 ± 1.03	0.14
	A2L	19.75 (6.80–26.67)	22.09 ± 2.43	19.52 ± 3.59	15.18 ± 3.70 ^abc^	< 0.01
	A2T	0.47 ± 0.20	0.60 ± 0.22	0.43 ± 0.16	0.45 ± 0.18	0.04
	A1L	8.27 ± 3.19	9.49 ± 3.94	10.27 ± 2.66	7.19 ± 1.88	0.01
	C1L	8.67 ± 2.74	9.38 ± 3.31	8.91 ± 2.44	7.74 ± 2.64^c^	0.33
	C2L	7.35 ± 2.32	8.30 (5.53–16.57)	7.69 (4.22–17.56)	6.12 (3.41–12.62)^b^	0.02
	C3L	6.25 ± 1.64	7.39 (4.61–16.43)	8.03 ± 2.42	5.70 ± 1.71^bc^	< 0.01
D. Morphometry of FDST radial and ulnar arm and FDPT	FDST-RW	2.71 ± 0.61	3.07 ± 0.73	2.25 (1.06–9.11)	1.74 ± 0.47^ab^	< 0.01
	FDST-RT	0.70 ± 0.22^b^	0.89 ± 0.22	0.73 ± 0.19	0.52 ± 0.17^bc^	< 0.01
	FDST-UW	2.97 ± 0.54	2.92 ± 0.69	2.62 ± 0.59	2.03 ± 0.58^abc^	< 0.01
	FDST-UT	0.70 (0.25–1.63)	0.88 ± 0.30	0.72 ± 0.22	0.40 ± 0.18^abc^	< 0.01
	FDPT-T	1.98 (1.46–3.20)	2.16 ± 0.59	1.96 ± 0.48	1.46 (1.06–4.69)^b^	0.04
	FDPT-W	4.88 (3.82–7.67)	5.48 ± 0.97	5.29 ± 0.99	4.11 ± 1.21^abc^	< 0.01
E. Location of chiasm, radial, and ulnar insertions point of the FDST	D1	16.87 ± 4.21	16.04 (7.15–19.99)	15.96 ± 3.31	12.73 ± 2.95^ac^	< 0.01
	D2	17.24 ± 4.10	15.91 ± 3.41	15.58 ± 2.98	12.73 ± 2.99^ab^	< 0.01
	D3	14.16 ± 3.70	14.03 ± 2.64	14.79 (11.61–26.58)	10.47 (7.67–18.74)^abc^	< 0.01
	D4	13.69 ± 3.61	14.30 ± 2.35	15.60 ± 2.69	11.27 ± 2.56^bc^	< 0.01
	D5	8.08 ± 3.12	9.09 ± 2.00	8.27 ± 1.64	6.99 ± 1.99	0.07
	D6	7.64 ± 1.89	9.18 ± 2.06	7.89 ± 1.89	7.45 ± 2.42	0.06
	D7	3.47 ± 1.22	3.82 (2.54–7.87)	3.44 (2.04–10.24)	3.31 ± 0.88	0.26
	D8	9.62 ± 3.19	9.84 ± 2.91	7.15 ± 1.56^ab^	7.12 ± 3.12^c^	< 0.01
	D9	5.58 ± 1	6.43 ± 0.97	5.69 ± 1.10	4.72 ± 0.89^c^	< 0.01
	H-FDST R	26.78 ± 4.50^b^	31.65 ± 5.04	28.81 ± 4.19	23.01 (18.44–34.41)^bc^	< 0.01
	H-C	10.95 ± 2.51	13.94 (9.13–22.43)	13.25 ± 3.45	11.66 ± 3.79	0.56
F. Relation of chiasm with A2 and A4 pulleys	A2D-H	9.31 ± 3.27	10.17 ± 5.02	9.75 (0.00–115.58)	9.66 (2.16–28.50)	0.34
	A2D-C	2.76 (0.00–11.13)	5.54 ± 4.26	3.93 ± 3.01	2.51 (0.00–21.60)	0.34
	A4P-H	27.77 (19.64–62.47)	32.90 (9.34–40.29)	29.35 ± 5.25	25.80 ± 7.30	0.18
	A4P-C	17.46 ± 3.76	18.76 14.62–37.15)	17.79 (12.19–34.38)	15.87 ± 4.94	0.08

^a,b,c^Difference in pairwise comparison by Bonferroni with fingers 2, 3, and 4 are marked as (^a^), (^b^), and (^c^), respectively (*n* = 20)

**Table 2:** Comparison (mm) of genders for all measurements [mean ± SD or median(25th–75th percentiles)]

		Male (*n* = 48)	Female(*n* = 32)
A. Relation of superficial landmarks (creases) with finger tip	FT-DPC**	24.98 ± 2.50	22.97 ± 1.60
	FT-PPC*	41.70 ± 4.68	38.49 (29.27–72.79)
	FT-PDC**	61.78 (34.62–75.77)	55.30 ± 7.02
	FT-IPC**	87.80 ± 10.79	78.05 ± 10.05
	FT-WC**	153.94 ± 15.81	139.80 (57.18–172.80)
B. Placement of pulleys according to fingertips	FT-A5D	17.74 ± 4.06	16.94 ± 2.38
	FT-A4D**	28.20 (17.33–33.94)	25.58 ± 3.48
	FT-A4P*	36.72 (23.33–41.84)	33.37 ± 3.78
	FT-A3D**	43.79 ± 5.29	39.34 ± 5.34
	FT-A2D**	56.84 ± 6.51	48.94 ± 6.69
	FT-A2P**	76.01 ± 9.46	68.22 (26.60–82.15)
	FT-A1P**	83.65 ± 11.38	73.81 ± 12.13
	FT-A1D**	78.34 ± 10.38	69.69 ± 7.96
C. Morphometry of pulleys	A4L*	8.04 (4.05–16.49)	7.96 ± 1.65
	A4T	0.41 (0.17–0.82)	0.42 ± 0.17
	A3L	2.98 ± 0.87	3.00 ± 0.90
	A2L**	20.12 ± 4.11	17.23 ± 3.84
	A2T	0.41 (0.13–0.91)	0.49 ± 0.18
	A1L	8.45 ± 3.08	9.34 ± 3.38
	C1L*	9.39 ± 3	7.60 ± 2.24
	C2L*	7.98 (3.41–17.56)	6.64 (3.75–16.57)
	C3L	6.41 (3.08–16.43)	6.80 ± 1.90
D. Morphometry of FDST radial and ulnar arm and FDPT	FDST-RW	2.53 ± 0.82	2.48 (1.06–9.11)
	FDST-RT	0.74 ± 0.25	0.67 ± 0.21
	FDST-UW	2.67 ± 0.70	2.59 ± 0.72
	FDST-UT	0.71 ± 0.32	0.63 (0.17–1.63)
	FDPT-T**	2.03 (1.18–4.69)	1.64 ± 0.40
	FDPT-W**	5.38 ± 1.12	4.40 ± 0.95
E. Location of chiasm, radial, and ulnar insertions point of the FDST	D1	16.07 ± 3.69	14.48 ± 3.46
	D2	16.14 ± 4.06	14.46 ± 3.03
	D3*	14.06 (8.86–26.58)	12.90 ± 2.98
	D4	14.28 ± 3.40	13.11 ± 2.84
	D5**	8.78 ± 2.44	7.22 ± 1.94
	D6	8.38 ± 2.47	7.53 (3.57–9.86)
	D7*	3.87 (1.64–10.24)	3.27 ± 0.84
	D8	8.33 ± 3.35	8.71 ± 2.52
	D9**	6.14 ± 1.03	4.90 ± 0.93
	H-FDST R	28.32 ± 5.07	27.34 ± 5.62
	H-C	13.55 ± 3.80	12.05 ± 3.65
F. Relation of chiasm with A2 and A4 pulleys	A2D-H	9.79 (0.00–115.58)	10.28 ± 4.35
	A2D-C*	4.39 (0.00–21.60)	2.28 (0.00–11.15)
	A4P-H*	30.61 (9.34–62.47)	27.41 ± 5.19
	A4P-C	18.38 (8.18–37.15)	16.99 ± 3.44

*p value < 0.05

**p value < 0.01

**Fig. 2:**
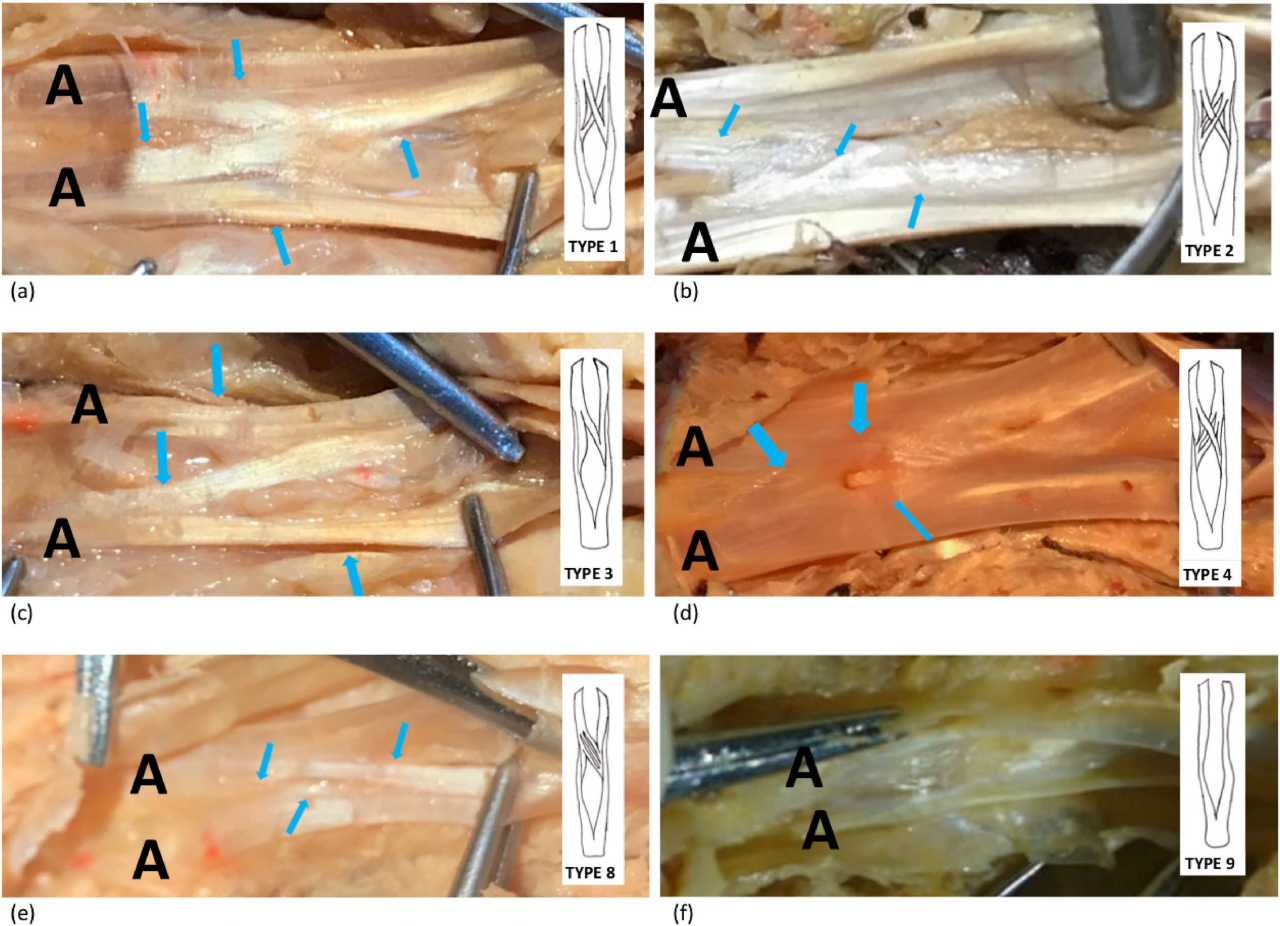
Demonstration of CT types. Blue arrows indicate fiber exchange. A: arm of chiasm, **a** chiasm type 1 (right 4th finger of a male), **b**: chiasm type 2 (right 3rd finger of a male), **c**: chiasm type 3 (right 5th finger of a male), **d**: chiasm type 4 (right 2nd finger of a male), **e**: chiasm type 8 (right 2nd finger of a female), **f**: absence of fiber exchange (type 9) (left 5th finger of a male)

**Fig. 3:**
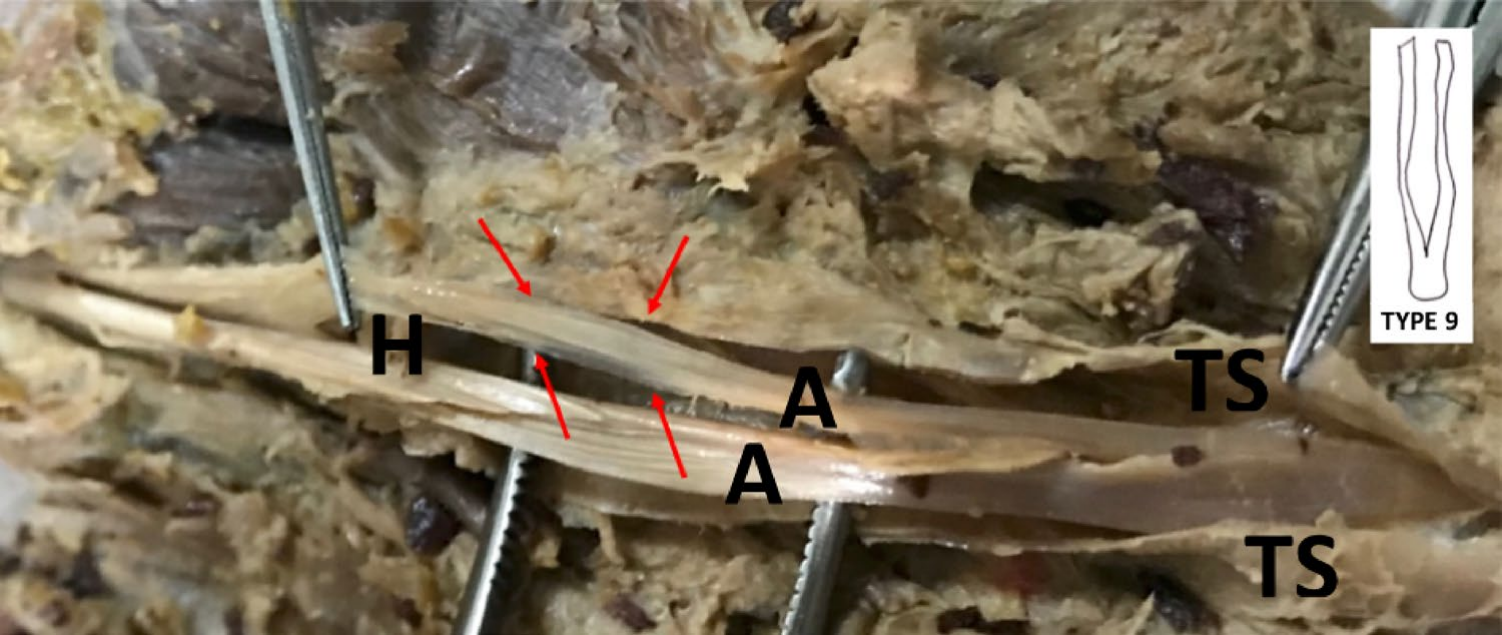
Unusually thin (pseudo)chiasm arm (right 5th finger of a male). H: tendinous hiatus, A: arm of chiasm. TS: longitudinally cut tendinous sheat

(a) Relations to superficial landmarks:

- Findings about the location of superficial landmarks (creases) to the fingertip were as follows: There was almost no difference in the superficial parameters among the 2nd, 3rd and 4th fingers (except comparison between 2nd and 3rd fingers in terms of the fingertip–interpalmar line distance) (Table 1). There was almost always a statistically significant difference between the values the 5th finger and that of other three fingers (except comparison between 2nd and 5th fingers in terms of the fingertip–distal interphalangeal crease distance). This finding might be a reflection of the similar lengths of the 2nd–4th fingers. In addition, in all of the superficial parameters, it was observed that the values were significantly greater in males than in females (*p* < 0.05) (Table 2).
- When the fingers were compared in terms of distance to the fingertip parameters for pulley and CT, significant differences were found most frequently between the 5th finger and the others; and less frequently, between the 2nd finger and the others. In all parameters, 3rd and 4th finger values were similar (*p* > 0.05). (Table 1).
- No significant difference was found for any parameter between the sides.

(b) Placement and morphometry of pulleys:

- Thickness of the A2 and A4 pulleys were similar between the fingers and in gender. In addition, the length of the A2 and A4 pulleys is significantly less in females than in males, similar for the 2nd, 3rd, and 4th fingers, while less in the 5th finger than in the others (*p* < 0.05) (Tables 1 and 2).
- The difference between genders was significant for the parameters about the distances of pulleys to the fingertip, the pulleys’ position on the finger, the length of the structures in CT region and length of the A2, A4, C1, and C2 pulleys (*p* < 0.05). However, there was no significant difference between the genders in parameters related to the width or thickness of the tendons and thickness of the pulleys.

(c) Morphometry of FDST and FDPT:

- FDST and FDPT morphometric parameters (i.e., width and thickness of FDPT and ulnar–radial arms FDST), values for 5th digit were significantly less than at least one of the fingers (*p* < 0.05); whereas values of the other fingers were mostly similar—except for one parameter (Table 1).
- Eleven of the 15 parameters of CT and FDST morphometry were similar in male and female. Parameters for distance between the distal point of the CT and distal point of the ulnar insertion, distance between the proximal and distal points of the ulnar insertion, distance between the two distal points of insertion and for width of the CT were significantly larger in male than in female. Some parameters related to CT morphometry (parameters regarding footprints of the radial and ulnar insertion of FDST, i.e., D5-D7) were similar among the fingers (Tables 1 and 2).

(d) Classification, location and relationship of CT with the pulleys:

- Pulley and CT positions were found to change according to finger size (FT-PDC column of the Table 3) (*p* < 0.01). The high correlation between pulley position parameters and superficial parameters can be expressed as the pulley position changes in parallel with finger length. Accordingly, in cadavers with longer fingers, pulleys are expected to be farther from the fingertip. The fact that thickness and length parameters of A2 pulley and A4 pulley do not correlate with superficial parameters and pulley position parameters on the finger is a finding that may be of surgical importance. The significant correlation between the width of the CT and the distance of both the proximal and distal borders of A2 pulley to the fingertip is also remarkable (*r* = 0.66, *r* = 0.63, respectively, *p* < 0.01)
- Frequencies of CT types with respect to fingers, sexes and sides were given in Table 4. Figure 1 shows the fiber exchange patterns corresponding to each CT type and the corresponding frequencies found in this study. Figure 2 (a-f) displays the well-exposed fiber exchanges and chiasm types of the study. Chiasm types with considerable bidirectional fiber exchange between the two branches of the FDST (types 1, 2, 4, 5, 6 and 7) as “symmetrical chiasm”, types with only one-sided fiber transfer from one arm to the other (types 3 and 8) as “asymmetric chiasm”, and the type where there is no fiber exchange between the two arms (type 9) was described as “pseudochiasm” (Fig. 1). The frequencies were found as 51%, 45%, and 4%, respectively.
- The fact that one of the two arms of the FDST in the 5th finger was very thin and shallow, which was seen in two cases, was noted as a variant case that may have clinical significance (Fig. 3).

**Table 3:** Correlation between the parameters for pulley position and position of the superficial landmarks by Pearson’s correlation test

Parameters	FT-DPC		FT-PPC		FT-PDC		FT-IPC		FT-WC	
	*r*	*p*	*r*	*p*	*r*	*p*	*r*	*p*	*r*	*p*
FT-A5D	0.27	0.02	0.30	< 0.01	0.35	< 0.01	0.25	0.02	0.39	< 0.01
FT-A4D	0.56	< 0.01	0.60	< 0.01	0.65	< 0.01	0.48	< 0.01	0.53	< 0.01
FT-A4P	0.61	< 0.01	0.60	< 0.01	0.67	< 0.01	0.45	< 0.01	0.48	< 0.01
FT-A3D	0.60	< 0.01	0.63	< 0.01	0.78	< 0.01	0.60	< 0.01	0.61	< 0.01
FT-A2D	0.54	< 0.01	0.57	< 0.01	0.74	< 0.01	0.54	< 0.01	0.59	< 0.01
FT-A2P	0.54	< 0.01	0.58	< 0.01	0.78	< 0.01	0.60	< 0.01	0.72	< 0.01
FT-A1P	0.47	< 0.01	0.60	< 0.01	0.74	< 0.01	0.58	< 0.01	0.60	< 0.01
FT-A1D	0.57	< 0.01	0.57	< 0.01	0.73	< 0.01	0.51	< 0.01	0.53	< 0.01

**Table 4:** Frequencies of chiasm types with respect to fingers, genders, and sides

	Type 1 (%)	Type 2 (%)	Type 3 (%)	Type 4 (%)	Type 5 (%)	Type 6 (%)	Type 7 (%)	Type 8 (%)	Type 9 (%)
Finger 2 (*N* = 20)	7 (35.00)	0 (0.00)	6 (30.00)	3 (15.00)	0 (0.00)	0 (0.00)	0 (0.00)	4 (20.00)	0 (0.00)
Finger 3 (*N* = 20)	10 (50.00)	0 (0.00)	7 (35.00)	1 (5.00)	0 (0.00)	1 (5.00)	1 (5.00)	0 (0.00)	0 (0.00)
Finger 4 (*N* = 20)	14 (70.00)	1 (5.00)	5 (25.00)	0 (0.00)	0 (0.00)	0 (0.00)	0 (0.00)	0 (0.00)	0 (0.00)
Finger 5 (*N* = 20)	0 (0.00)	1 (5.00)	13 (65.00)	2 (10.00)	0 (0.00)	0 (0.00)	0 (0.00)	1 (5.00)	3 (15.00)
Male (*N* = 48)	16 (33.33)	2 (4.18)	21 (43.75)	4 (8.33)	0 (0.00)	1 (2.08)	1 (2.08)	1 (2.08)	2 (4.17)
Female (*N* = 32)	15 (46.88)	0 (0.00)	10 (31.25)	2 (6.25)	0 (0.00)	0 (0.00)	0 (0.00)	4 (12.50)	1 (3.12)
Right (*N* = 40)	12 (30.00)	2 (5.00)	19 (47.50)	3 (7.50)	0 (0.00)	1 (2.50)	0 (0.00)	2 (5.00)	1 (2.50)
Left (*N* = 40)	19 (47.50)	0 (0.00)	12 (30.00)	3 (7.50)	0 (0.00)	0 (0.00)	1 (2.50)	3 (7.50)	2 (5.00)
Total (*N* = 80)	31 (38.75)	2 (2.50)	31 (38.75)	6 (7.50)	0 (0.00)	1 (1.25)	1 (1.25)	5 (6.25)	3 (3.75)

## Discussion

Our findings about the morphometric details of the three layers (the superficial landmarks (i.e., palmar digital creases and fingertip), the tendon sheath (pulleys) and the flexor tendons) on the palmar side of each finger, as well as the findings about the relationships between the layers, differences among the fingers and between the genders, were evaluated in terms of contribution to hand surgery.

Clinical relevance of CT is especially prominent in zone 2 (and occasionally zone 1) hand surgeries. Repair of acute flexor tendon injuries and flexor tendon reconstruction are major examples of such surgical approaches [21]. It is recommended that pulleys are preserved during surgery, or reconstucted to avoid cases, such as “bowstringing” [1, 3, 6, 10, 15, 20, 21]. One arm of the CT is used to reconstruct pulleys [21]. FDST lacerations distal to CT are more challenging to repair since each tendon attachment is notably weak [21]. The morphometric properties of CT, presented in detail in this study, may contribute to such surgical procedures.

Different pulley positions or lengths could require to change primary incision location or to revise the surgical procedure. In this regard, pulley-related morphological comparisons between fingers and genders may be an additional input for hand surgeons, whereas studies comparing genders are very limited in the literature. There have been many studies on the pulley length, which is attributable to its importance in hand surgery. The pulley length studies present in the databases scanned and comparable with this study, focused mainly on A1 pulley. Grinčuk et al. measured A1 pulley length in 14 fresh-frozen cadavers with needle palpation method and reported the following values; 5.0, 5.0, 4.1, and 3.7 mm for 2nd to 5th fingers, respectively [9]. The boundary between A1 pulley and A2 pulley, which are very close to each other, can be difficult to distinguish without direct exposure. Therefore, it is possible that the reason their measurements are smaller than in ours might be due to the differences in methods—i.e. dissection vs needle palpation, rather than geographic origin differences.

Mallo et al. studied biomechanical properties of A2 and A4 pulleys between genders for 2nd to 5th fingers and concluded that the differences are not statistically significant [13]. No significant difference was found in our study for A2 and A5 pulley thickness among the fingers, and the lengths of the A2 and A4 pulleys are similar for the 2nd, 3rd and 4th fingers (5th finger was smaller than the others). Those could be the underlying morphological reason behind biomechanics of A2 and A4 pulleys reported in the study of Mallo et al. [13]. However, the fact that the A2 and A4 pulley lengths are significantly smaller in female than in male in our study contradicts with Mallo et al. [13].

Identifying relations between superficial landmarks and deeper structures, and variations in pulley position could be helpful for planning flexor tendon and tendon sheath surgeries. Fiorini et al. measured A1 pulley (proximal edge) to metacarpophalangeal crease distance, A1 pulley length and metacarpophalangeal crease to proximal interphalangeal crease distance for 2nd to 5th fingers, and their findings are in accordance with ours [5]. The findings of Watkins et al. for A1 pulley (proximal edge) to interpalmar crease distance are also similar to ours, except for the 3rd finger—which may be due to an uncertainty in the definition of interpalmar crease, which particularly concerns the level of 3rd finger [22].

Gordon et al. conducted a study, to estimate the pulley positions and determine optimal incision areas [8]. They used predetermined superficial landmarks and relative positions instead of having a unique reference point (e.g. fingertip). Mayhew et al. found finger lengths and hand width to be less in females than in males [14], shorter finger length in females was in line with the findings of this study.

In our study, it is found that the pulley position changes in parallel with finger length, and in the longer fingers, pulleys are farther from the fingertip. The fact that the thickness and length parameters of A2 pulley and A4 pulley do not correlate with superficial parameters and pulley position parameters on the finger is a finding that may be of surgical importance. On the other hand, the lengths of the A2 and A4 pulleys are similar for the 2nd, 3rd, and 4th fingers, while the 5th finger is smaller than the others, and they are significantly smaller in females than in males. The significant correlation between the width of the CT and the distance of the A2 border to the fingertip is also a remarkable finding of this study. The average values and determinations revealed in this study for each finger and gender regarding pulleys can help in predicting the pulley position with better precision.

The differences observed in the frequencies of chiasm types in the studies of Schmidt et al. and Gonzalez et al. point to the abundance of variations in chiasm. Our study and Gonzalez et al.’s results have similar findings, such as Type 1 was the most frequent chiasm type for 2nd, 3rd, and 4th fingers. (37.5%, 57.5%, and 55% for fingers 2, 3, and 4, respectively, in Gonzalez et al.’s study; 35%, 50%, and 70% for 2nd, 3rd, and 4th fingers, respectively, in our study). On the other hand, Gonzalez et al. reports the Type 1 in the 5th finger as 22.5%, but we did not encounter Type 1 in the 5th finger in our series. Type 4 and Type 10 in the Gonzalez’s classification were not encountered in our study and also in the study of Schmidt et al. We think that contradictions cannot be explained solely by geographic origin differences that too many variations of the chiasm may be responsible, and studies with larger series are needed. [7, 18]

Pike et al. assumed chiasm to be of symmetrical morphology and thus expected there to be no net torque in flexion in coronal plane [17]. In this study, the asymmetrical types were observed to be frequent enough to reach 45% (type 3 as 38.75% and type 8 as 6.25–45.00% in total), which contradicts the assumption of Pike et al. and might have clinical implications [17]. Asymmetrical chiasm types were observed in less frequency in Schmidt et al.’s (19% in total) and Gonzalez et al.’s (16% in total) study [7, 18].

Type 9 (no fiber exchange) was observed only in the 5th finger in our study (in 3 out of 20 5th fingers). This might be a risk factor contributing to vulnerability of the fifth finger. Additionally, some 5th finger chiasm arms were observed to be much thinner than other fingers, sometimes to the degree of being partially transparent. Figure 3 is an example of such a case. Ulnar arm of the 5th finger FDST was found to be narrower and thinner than 2nd, 3rd, and 4th fingers; radial arm was found to be narrower than 2nd and 3rd fingers and thinner than 3rd and 4th fingers. Li et al. reported FDPT rupture in 5th finger without injury in a case report. They considered chiasm to have a function similar to pulleys and increase FDPT elasticity [12] which might in conjunction with our type 9 chiasm findings explain spontaneous injury in flexor tendons of 5th finger.

In earlier review about the tendon rupture, Boyes et al. (1960) suggested the actual spontaneous ruptures to be seldom (3 of 80 cases) [3]. Fourth finger tendon rupture cases were found by Boyes et al. as the most frequent (30%) and the fifth finger rupture cases were found as 16%. Boyes et al. suggested such ruptures to be often followed by some kind of specific vulnerability, that were in most of the cases injuries, bone anomalies, or diseases. However, in a more recent study on 50 spontaneous flexor tendon ruptures, Bois et al. found 62% of the spontaneous ruptures in 5th finger and blamed underlying conditions, such as vascular insufficiencies, trauma, and anatomic variations [2]. Bois et al. emphasized more proximal variations (e.g. at insertion of lumbricals, at wrist), but did not mention chiasm variations. Lee et al. focused on 5th finger flexor tendon ruptures in 104 cases and concluded that truly spontaneous cases to be less frequent than reported [11]. Lee et al. blamed joint pathologies, tendon anomalies and similar conditions, while regarded hypovascularity to be irrelevant [11]. Although disagreement about the definition of “spontaneous” flexor tendon rupture was evident, 5th finger flexor tendon rupture was clearly reported as frequent in all of the three studies. In this study, type 9 chiasm (no fiber exchange) was found only in 5th finger and the arms of FDS was the thinnest and narrowest, which might be a risk factor for the high tendon rupture frequency of the 5th finger. Of course, further studies with clearer evidence are needed to confirm whether the structural features described for the 5th finger play a role in tendon rupture.

The limitation of the study is that although there are many variations in each structure in the region, the number of cadavers is limited. To generalize the statistically significant results obtained from such a limited number with comprehensive morphometric evaluations, studies with larger series taking into account age and geographic origin differences are needed.

## Conclusions

The morphometric properties of the three layers of each finger (creases, pulleys, and CT) and their relationships that are revealed, may be helpful in operations requiring manipulation of flexor tendons and in interpreting tendon ruptures. Asymmetric chiasm types were 45% in this study, wether they have tortion effect can be tested by future biomechanical studies in fresh frozen cadavers.
